# Contributions of Multilevel Family Factors to Emotional and Behavioral Problems among Children with Oppositional Defiant Disorder in China

**DOI:** 10.3390/bs13020113

**Published:** 2023-01-29

**Authors:** Ting He, Jocelyn Meza, Wan Ding, Stephen P. Hinshaw, Qing Zhou, Umair Akram, Xiuyun Lin

**Affiliations:** 1 School of Developmental Psychology, Faculty of Psychology, Beijing Normal University, Beijing 100875, China; 2 Department of Psychology, University of California, Berkeley, CA 94720, USA; 3 Department of Psychology, Teacher Education College, Zhejiang Normal University, Jinhua 321004, China; 4 Hangzhou College of Commerce, Zhejiang Gongshang University, Hangzhou 310018, China; 5 Beijing Key Laboratory of Applied Experimental Psychology, Faculty of Psychology, Beijing Normal University, Beijing 100875, China

**Keywords:** behavioral problems, Chinese children, emotional problems, multilevel family factors, oppositional defiant disorder

## Abstract

Oppositional defiant disorder (ODD) is one of the most prevalent childhood mental health disorders and is extremely affected by family factors. However, limited studies have addressed the issue from the perspective of family systems. The current study examines the associations between multilevel family factors (i.e., family cohesion/ adaptability at system level, mother–child and father–child attachment at a dyadic level, and child self-esteem at an individual level) and emotional and behavioral problems among children with ODD in China. The participants were 256 Chinese children with ODD and their parents and class master teachers. A multiple-informant approach and structural equation model were used. The results revealed that system level factors (family cohesion/adaptability) were associated with child emotional and behavior problems indirectly through factors at the dyadic level (mother–child attachment) and the individual level (child self-esteem) in sequence. Mother–child, but not father–child, attachment, mediated the linkage between family cohesion/adaptability and the emotional problems of children with ODD. Moreover, child self-esteem mediated the association between mother–child attachment and child emotional and behavioral problems. The findings of the present study underscored that multilevel family factors are uniquely related to emotional and behavioral problems in children with ODD.

## 1. Introduction

Oppositional defiant disorder (ODD) is characterized by a recurrent pattern of angry/irritable mood, argumentative/defiant behavior, and vindictiveness toward authority figures or adults as well [1]. Previous findings indicated that children with ODD have comorbid emotional and behavioral problems, such as depression and aggressive behavior [2]. Indeed, 45.8% of those with a lifetime diagnosis of ODD met the criteria for depressive disorder [3], and depression was a key contributor to behavioral problems in childhood [4]. Additionally, childhood ODD has been associated with an increased risk of conduct disorder ([CD]; [5]), which has a high probability of developing antisocial personality disorder in adulthood [1]. Due to the significant risk that emotional and behavioral problems pose to adjustment in typically developing children [6,7], children with ODD who have comorbid emotional and behavioral problems might be at a much higher risk for child outcomes [1,8,9]. Therefore, it is necessary to examine the influential factors on emotional and behavioral problems in children with ODD. Studies examining these links would deepen our understanding of ODD and have prominent implications for developing effective intervention programs.

Several family risk factors contribute to the severity of emotional and behavioral problems of children with ODD [10,11]. Notably, Lin and colleagues (2022) proposed a multilevel (i.e., system, dyadic, and individual level) family factors model to illustrate the associations between family factors at different levels and child ODD symptoms [12]. Regarding the system level, family is considered as a complete unit and system consisting of surface characteristics (e.g., social–economic status) and deep characteristics (e.g., family function). The dyadic level refers to the functioning of each subsystem in the family, including the wife–husband subsystem and parent–child subsystem. The individual level considers each family member as a separate subsystem. According to Lin et al.’s model, family factors at different level were associated with child ODD symptoms uniquely. However, it remained unclear exactly how multilevel family factors were, respectively, associated with co-occurring emotional and behavioral problems. To enrich the multilevel family factors model, we constructed a comprehensive model to examine the associations between multilevel family factors and emotional and behavioral problems of children with ODD. Specifically, we considered family cohesion/adaptability at a system level, mother–child and father–child attachment simultaneously at dyadic level, and child self-esteem at an individual level. Identifying modifiable family factors for emerging problem behaviors is necessary for family-based education and intervention programs.

### 1.1. Factor at System Level Associated with Emotional and Behavioral Problems in Children with ODD

At the system level of family environment, researchers have underscored the contribution of family function to the emotional and behavioral problems of children with ODD [13]. Olson (2000) pointed out that family cohesion and adaptability are two core components of family function. Family cohesion refers to the emotional connection among family members [14,15], while family adaptability refers to the ability of a family system to change its power structure, role relationships, and relationship rules in response to situational and developmental stress [14]. According to the McMaster family functional model theory [16], a poor family function might lead to less open communication and a steady accumulation of negative emotions. Children in such a family environment would gradually learn the negative interaction pattern, which increases the risk of physical diseases and problem behaviors. Empirical research has been carried out on the role of family cohesion/adaptability in child problem behaviors as well. For example, Lavigne and colleagues (2012) found that family cohesion/adaptability attenuated the risk of emotional and behavioral problems in children with ODD. On the contrary, family conflict appeared to facilitate emotional and behavioral problems in children [11].

### 1.2. Factor at Dyadic Level Associated with Emotion and Behavioral Problems in Children with ODD

Regarding the dyadic level, studies were inclined to explore dysfunctional parent–child interactions independent of other family-related interactions [17,18]. Research concerning attachment theory has revealed that parent–child attachment was associated with children’s problem behaviors [6]. Attachment theory implies that parent–child attachment is prominently linked to child problem behaviors by shaping the internal work model of the child. If the caregivers are lacking in sensitivity or are even frightening to the children, children would be insecurely attached and more likely to develop a maladjusted internal work model [19]. Repeated experiences of the insensitivity of the caregiver would lead to dysfunctional cognition about the self and others and negative expectations in interpersonal interactions, which might enhance the risk for problem behaviors. Empirical studies consistently indicated that a lower level of parent–child attachment was associated with more emotional and behavioral problems in children [20,21]. Similar findings have been reported for children with ODD as well [10].

Attachment theory asserts further that children could form multiple attachments, i.e., they might develop distinct attachment with their mothers and fathers [22]. As such, it is important to point out that researchers have emphasized the need to separate the father and mother when examining their respective contributions to child development [7]. For one thing, mothers and fathers play different caregiving roles in families and have distinct interaction patterns with children. Therefore, mother–child and father–child attachments might associate with their children’s development uniquely [23]. According to the dominant hypothesis [24], a child’s attachment to his or her mother plays a pivotal part in his or her psychological development, due to the fact that the mother and child have more opportunities to spend time together [25]. According to the specificity hypothesis [26], the attachment a child forms with either his or her mother or father has distinct effects on his or her development [27]. Furthermore, during middle childhood, mothers and fathers are inclined to interact with children separately, increasing the opportunities for mother–child and father–child attachment to play different roles in child development [28]. However, mixed findings have emerged regarding the links of mother–child and father–child attachment with problem behaviors in children. Specifically, some have argued that mother–child attachment was closely related to child emotional problems [29], while Pan et al. (2016) proposed that father–child attachment was more crucial than mother–child attachment in predicting psychological health in Chinese children. However, Carter (2014) argued that secure attachments with both mothers and fathers protected children from worse emotional symptoms [30]. We do not yet have a full understanding of how mother–child and father–child attachments predict emotional and behavioral problems in children with ODD uniquely. Therefore, this study intends to distinguish the two different parenting roles and discuss them separately.

### 1.3. Factor at Individual Level Associated with Emotion and Behavioral Problems in Children with ODD

With regard to individual level factors, according to the multilevel family factors model and the research findings, several individual child factors are associated with the development of child ODD symptoms, such as child individual characteristics (children’s temperament), cognitive factors (social cognition), and emotion-related factors (emotion regulation), etc. [12,31,32]. This research focused on child self-esteem, which has been found to play a predictive role in co-occurring emotional and behavioral problems. Given that children with ODD frequently receive negative social feedback throughout their development, they were more likely to experience lower levels of self-esteem [8,9]. Based on the self-esteem theory of depression, low self-esteem is one of the most important susceptibility qualities for depression [33] and the social bonding theory points out that low self-esteem contributes to less consistency in social norms and more problem behaviors [34]. Other studies have also revealed that child self-esteem is linked to the overall outcomes of children, including emotional and behavioral problems. For instance, a longitudinal study conducted by Leeuwis and colleagues (2014) indicated that low self-esteem was a strong predictor of subsequent internalizing symptoms in children [35]. Lin and colleagues (2014) also found that lower self-esteem was associated with higher levels of depression and more aggressive behaviors in children with ODD [36]. However, less is known about the role of child self-esteem in the emotional and behavioral problems of children with ODD in the family context. As such, the present study aims to explore how child self-esteem, as an individual level factor, is related to other family factors at system a level and a dyadic level, and ultimately to child emotional and behavioral outcomes.

### 1.4. Interplay among Factors at Three Level

According to the person–context interaction theory [37], the environmental factors vary from distal to proximal and the processes of interplay between the distal and proximal environmental factors affect the development of an individual. Magnusson and Stattin (1998) further indicated that the distal factors decide the opportunities and restrictions for the functioning and development of proximal factors, as well as the individuals. In the family system, family function is a distal environmental factor, parent–child attachment is a proximal environmental factor, and child self-esteem is the most proximal factor for children [12,38]. As such, family function might directly and indirectly predict child problem behaviors via the parent–child relationship and the child’s self-esteem. Additionally, the parent–child relationship might directly and indirectly predict child problem behaviors via the child’s self-esteem.

Indeed, families with poor function tended to have poor communication, which would lead to less parent–child interaction and a lower level of parent–child attachment. Consequently, children might be likely to form maladaptive internal work models and develop low self-esteem. All of these might cause or exacerbate problem behaviors in children. Some preliminary empirical evidence supported these assumptions. For instance, the effect of family cohesion/adaptability on child depression is likely to be mediated by parenting (dyadic level), parental depression, and child temperament (individual level) [39]. Liu and colleagues (2018) found that child self-esteem mediated the spillover effects between family cohesion/adaptability and the emotional problems of children [40]. Additionally, existing evidence supports that child self-esteem serves as a mechanism explaining the link between parent–child attachment and the emotional and behavioral problems of children [41,42]. However, it remains unknown whether the processes of interplay among three different system levels could be associated with emotional and behavioral problems in children with ODD uniquely. In the current study, we include family cohesion/adaptability at a system level, mother–child and father–child attachment simultaneously at a dyadic level, and child self-esteem at an individual level to explore the role that family factors at different levels play in the emotional and behavioral problems of children with ODD.

### 1.5. Influence of Chinese Culture

Since the cultural context affects both the whole family and the individual family member, it is essential to comprehend the associations between family factors and child outcomes in the cultural context. In various aspects, Chinese culture differs from Western culture. First, Chinese parents are typically more involved in their children’s upbringing than American parents [43]. Under the influence of Confucianism, Chinese society adopted hierarchical parent–child interactions and disciplinarian parental socialization [43], which may contribute a substantial parental effect on child development.

Second, it should be noted that most children in the present study were the only-child in their families. As the only child, some families adopted a “child-centered” parenting style [44]. While this parenting style might improve the quality of parent–child attachments, it might also increase the emotional and behavioral problems in children [45]. For one thing, a child-centered approach means that parents might spoil their children and fail to discipline children’s daily behaviors, which increases the risk of behavior problems. For another, a child-centered approach would make parents place high expectations on their children and expect them to achieve excellent school performance, while paying less attention to their children’s psychological needs. All of these factors might increase children’s emotional and behavioral problems.

Third, there is a well-known expectation in some Asian, African, or economically underdeveloped countries, “men outside the home, women inside (*Nan Zhu Wai, Nv Zhu Nei*)”, because of traditional gender roles, and China is one of them [46]. Traditionally, Chinese mothers tend to take on full caregiving responsibilities in the household, while fathers are responsible for providing the financial necessities for the household. This division of household labor may contribute to a closer bond between children and their mothers than their fathers. Additionally, Chinese society expects married women to be “good wives and mothers (*Xian Qi Liang Mu*)”, while the men are expected to “earn money to support the family”. The different cultural expectations make the mothers devote more time and energy to raising children than the father. Therefore, Chinese mothers may have a greater influence on their children’s socio-emotional development.

In contemporary Chinese families, however, parental notions on the parent–child attachment and its influence on children are shifting [47]. Modern Chinese mothers demonstrate less closeness and connection with their children compared with mothers of the previous century [47]; fathers play a less “authoritarian” role in the family and have more intimate interaction with children [48]. Nevertheless, it is plausible that traditional and contemporary parenting practices coexist in Chinese households, and it is unknown how the effects of parent–child attachment on children’s development differ depending on the role of the parent.

### 1.6. The Present Study

**Figure 1:**
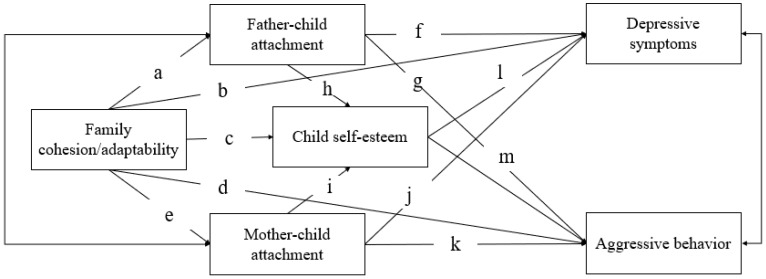
Proposed Model of Multilevel Family Factors and Emotional and Behavioral Problems of Children with ODD. **Note.** a—m indicates the effects of multilevel family factors on child depressive symptoms and aggressive behavior.

The current study examined how multilevel family factors were differently related to emotional and behavioral problems in Chinese children with ODD. Specifically, we included family cohesion/adaptability as the system level factor, mother–child and father–child attachment concurrently as the dyadic level factors, and child self-esteem as the individual level factor (see Figure 1 for the proposed model). Three problems would be explored: (a) whether family cohesion/adaptability is significantly related to the emotional and behavioral problems of children directly or indirectly through both dyadic level (mother–child and father–child attachment) and individual level (self-esteem of children) factors; (b) whether child self-esteem would mediate the linkages between mother–child and father–child attachments and emotional and behavioral outcomes; (c) whether the mother–child attachment would be more closely linked to the emotional and behavioral problems of children rather than the father–child attachment.

Based on the theoretical backgrounds and empirical research, three hypotheses were proposed: (a) family cohesion/adaptability is significantly related to the emotional and behavioral problems of children directly or indirectly through both dyadic level (mother–child and father–child attachment) and individual level (self-esteem of children) factors, (b) child self-esteem would mediate the linkages between mother–child and father–child attachment and emotional and behavioral outcomes, and (c) mother–child attachment would be more closely linked to the emotional and behavioral problems of children than father–child attachment.

## 2. Method

### 2.1. Procedure

There were six primary steps in the recruitment process. First, to obtain the informed consent of schools. Using a convenience sampling method, we reached out to the primary principals and school psychologists of 20 cooperative primary schools and invited them to participate in this study. Of these schools, 14 elementary schools in Beijing (8), Shandong Province (2), and Yunnan Province (4) agreed to participate. The three areas are located in the North, East, and Southwest of the mainland and represent developed, developing, and undeveloped regions in China. All 14 of these primary schools are day schools, four are priority primary schools, and thirteen are situated inside the city. The range of pupils was between 300 and 5000. (2 schools have fewer than 1000 students and 7 schools have more than 2000 students). We first obtained the consent of the principals and school psychologists of these schools.

Second, to obtain the informed consent of class master teachers. We asked the school psychologists to issue research invitations and informed consent forms to class master teachers of grades first through fifth. Eventually, 187 class master teachers signed informed content and agreed to participate in our study.

Third, nomination. These 187 class master teachers were asked to nominate the children who might have ODD symptoms in their classes, according to the eight-item ODD assessment checklist (DSM-IV-TR, 2000), children who displayed four or more symptoms for at least 6 months with damaged relationship functions were nominated.

Fourth, confirmation. Two clinical psychologists from the research team interviewed each participating class master teacher to confirm the accuracy of the nomination. The confirmation criterions were based on DSM-IV-TR diagnostic criteria: (a) elementary students in grades one through five; (b) the child shows four or more symptoms of ODD; (c) the identified ODD symptoms have lasted for six months or more; (d) the child exhibits serious impairment across psychosocial functioning domains; and (e) without intellectual disability and other disorders, such as dyslexia, autism spectrum disorder, etc. Only children with both clinical psychologists’ diagnoses of ODD were recruited into this study. Eventually, 305 children were identified to have ODD of the total 7966 children.

Fifth, to obtain the informed consent of parents. Invitation letters and informed consent were sent to 305 parents of children identified with ODD symptoms. A total of 282 pairs of parent and child gave informed consent and assent forms were obtained (92.5% participation rate).

Finally, these 282 children were asked to forward a package containing a parent survey to his or her primary caregiver. The primary caregiver (either the mother or the father, each family decide for themselves according to the reality of raising children) were invited to fill out the survey and to return their completed surveys to the class master teacher within one week. After parents signed informed consent, children completed the student questionnaire in a school conference room or music room, while trained researchers stayed in the room to provide assistance and explain the meaning of sentences when necessary. Specifically, children in grades 3, 4, and 5 were supervised by one teacher and one clinical psychology researcher. Due to the possibility that children in Grades 1 and 2 (ages 6–7) might have trouble comprehending the questionnaire, four to five teachers and researchers were assigned to guarantee that they could assist each child individually if children had difficulty completing questions. Both survey methods required children to independently complete questionnaires; the only difference was the number of researchers. Previous research has shown that the findings of a self-administered survey and an individual interview are compatible and comparable [49]. Class master teachers were also invited to complete a questionnaire to assess the behavior of each child in the study. A total of 256 parent–child dyads completed data collection, which included at least one of their parents and class master teachers’ finished questionnaires.

Prior to conducting the study, the Institutional Review Board of [mask for review] University in China approved the research protocol, including the consent procedure [Approval number]. We obtained active consent from parents, students, and teachers prior to data collection, and we promised to keep the participants’ information confidential. For interested parents of the identified children, psychiatrists from Anding Hospital, mental health counselors, and a family therapist from the Center of Family Study and Therapy at [mask for review] University offered opportunities for ODD treatment.

### 2.2. Participants

The final ODD sample consisted of 256 parent–child dyads, including 83 father–child dyads and 173 mother–child dyads. The participating children included 186 boys and 69 girls, with 1 missing gender information. Among these children, 75.8% were the only child in family. Fathers’ ages ranged between 25 and 54 years (M = 38.43, SD = 5.16), and mothers’ ages ranged between 26 and 53 years (M = 36.66, SD = 4.29), and children’s ages ranged between 6 and 13 years (M = 9.60, SD = 1.57). Regarding educational level, most mothers (56.6%) and fathers (61.6%) had junior college diplomas or above. For family social economic status, 56.3% families had a monthly income over 5000 Chinese Yuan (approximately $720; the average monthly income for Chinese urban families is about 5485 Chinese Yuan in 2015; [50]).

### 2.3. Measures

#### 2.3.1. ODD Symptoms

Class master teachers, school psychological teachers and two clinical psychologists were asked to assess children’s ODD symptoms based on the 8-item diagnosis of ODD scale in DSM-IV-TR (0 = no; 1 = yes; e.g., “often loses temper”, “often argues with adults”; [1]). Children who had four or more items of the 8-item scale were identified with ODD. Scores were summed across the eight items and higher sum scores indicated that the child exhibited more ODD symptoms. The Cronbach’s α was 0.85 in the current study.

#### 2.3.2. Family Cohesion/Adaptability (Parent Reported)

The family cohesion/adaptability was assessed by the *Family Adaptability and Cohesion Evaluation Scale* (FACES-II; [14]), which has been validated as an appropriate measure for use in China [51]. FACES-II assesses the family function in two dimensions: adaptability (14 items; e.g., “In solving problems, the children’s suggestions are followed”) and cohesion (16 items; e.g., “Family members like to spend free time with each other”). The correlation coefficient between the adaptability and cohesion was 0.78 (*p* < 0.001) in the current study. Each parent reported their perception of the family function using a 5-point Likert scale 1 = almost never to 5 = almost always). A composite score was created by summing the scores of two dimensions. A higher total of scores on FACES-II indicated better adaptability and cohesion in the family. In the current study, the Cronbach’s α for FACES-II was 0.84. Additionally, given that the data of family cohesion/adaptability was collected from either a father or a mother, an independent t-test was conducted to compare fathers’ reports and mothers’ reports. The result indicated that there was no significant difference in fathers’ and mothers’ report of family cohesion/adaptability (*t* = −0.23, *p* > 0.05).

#### 2.3.3. Parent–Child Attachment (Child Reported)

Parent–child attachment was measured by child report of the Chinese Version of parent subscales *of the Inventory of Parent and Peer Attachment* (IPPA; [52,53]). This measure and its subscales have been demonstrated as having acceptable construct validity and internal consistency in a sample of Chinese primary school-aged children [54]. Each child was asked to rate their attachment to both mother and father on the following dimensions: trust (5 items; e.g., “‘My father/mother respects my feelings”); communication (5 items; e.g., “If my father/mother knows something is bothering me, he/she asks me”); and alienation (5 items; e.g., “I am angry with my father/mother”), with parallel wordings of items for assessing relationships with mothers and fathers. All items are rated on a 5-point frequency response scale ranging from 1 (almost never) to 5 (almost always). A composite score was created for each mother–child and father–child attachment by subtracting the scores of alienation subscale from the sum scores of trust and communication subscales [53]. The higher scores indicated higher levels of parent–child attachment. In the current study, the Cronbach’s *α* was 0.88 for both mother–child attachment and father–child attachment, respectively.

#### 2.3.4. Child Self-Esteem (Child Reported)

Child self-esteem was assessed using the *Self-Esteem Scale* (SES; [34]. The scale was shown to be a reliable and valid measurement for elementary school-aged children in China [49]. Each participating child reported on their own self-esteem by using a 4-point scale (1 = strongly disagree to 4 = strongly agree) on 10 items (e.g., “I am a person of worth”). A reversed scoring was used for the five items with negative states. Scores were summed to create a composite score, the higher score indicated higher levels of self-esteem. The Cronbach’s α was 0.84 in the current study.

#### 2.3.5. Children Depressive Symptoms (Child Reported)

Children’s self-report of depressive symptoms were assessed using the *Center for Epidemiological Studies Depression Scale for Children* (CES-DC; [55]), Researchers have validated the CES-DC for the assessment of depressive symptoms in Chinese children [56]. The CES-DC consists of 20 items (e.g., “I was bothered by things that usually don’t bother me”) and each item was rated on a 4-point scale (1 = not at all to 4 = a lot). Summed scores were used as a measure of child depressive symptoms, with higher scores indicating more severe depressive symptoms. The Cronbach’s α of this study was 0.86.

#### 2.3.6. Aggressive Behavior (Teacher Reported)

Child aggression was measured using the “*Aggressive with Peers*” subscale from the Child Behavior Scale (CBS; [57]). Previous study has proved this scale for measuring child aggressive behavior by teacher [58]. Class master teachers rated each child’s aggressive behavior toward peers by using a 5-point scale (1 = never to 5 = always) on 7 items (e.g., “This child pushes or shoves other children”). Scores were summed to create a composite score, with a higher score indicating more aggressive behavior towards peers in school. The Cronbach’s α was 0.96 in the current study.

### 2.4. Data Analysis

Preliminary data analyses were performed using SPSS 20.0. and Mplus 7.0. First, given that the data of family cohesion/adaptability was collected from either a father or a mother, the multiple group analysis was implemented in Mplus 7.0 [59] to examine the possibility of a gender difference among reporters. An unconstrained model that allowed the 13 paths (i.e., path a-m, see the Figure 1) estimates to vary among father-report and mother-report group was estimated. This model fit the data well, χ^2^ (42) = 41.30, CFI = 1.00, RMSEA = 0.00, SRMR = 0.05. Next, a constrained model that constrained the parameter estimates of 13 paths for the father-report and mother-report group to be equal was estimated. If this constrained model resulted in a statistically significant decrement of model fit (χ^2^) in comparison with the unconstrained model, then the pattern of associations could be assumed to vary for the father-report and mother-report groups. This model revealed a good fit for the data, χ^2^ (55) = 54.65, CFI = 1.00, RMSEA = 0.00, SRMR = 0.06. Results indicated that the model constraining the 13 paths coefficients to be equal across the two groups did not fit significantly worse than the model with these 13 path coefficients freely estimated across groups (Δχ^2^ = 13.35, Δ *df* = 13, *p* = 0.42), suggesting that the current model did not differ across gender of reporters. Therefore, our study did not distinguish them in the model.

Then, descriptive statistics were performed using SPSS 20.0 on all demographic variables (i.e., child gender, child age, educational years of parents, and family monthly income) and observed variables (i.e., family cohesion/adaptability, mother–child and father–child attachment, child self-esteem, depressive symptoms, and aggressive behavior).

After that, the simple Pearson’s correlations between observed and demographic variables were computed in order to understand relations between them.

Primary analyses were conducted with the structural equation model (SEM) within Mplus 7.0. The proposed multiple mediation model (see Figure 1) with covariates (i.e., child gender, child age, educational years of parents, and family monthly income) was examined to test for possible mediation effects. The fit indices used to evaluate the model were the chi-square statistic (*χ*^2^), goodness-of-fit index (CFI), Tucker–Lewis index (TLI), root mean square error of approximation (RMSEA), and standardized root mean residual (SRMR). Model fit was considered acceptable when the values of χ^2^ were not significant, and CFI > 0.95, TLI > 0.95, RMSEA < 0.08, and SRMR < 0.08 [60]. A bootstrapping procedure with 5000 iterations was used to test the indirect effects, in which a 95% confidence interval (CI) excluding zero indicates a significant mediating pathway. Missing data were addressed using Mplus’ default of the full information maximum likelihood method (FIML) [61].

## 3. Results

### 3.1. Descriptive Statistics among All Variables of Interest

Descriptive characteristics and the correlations among study variables are presented in Table 1. Family cohesion/adaptability, father–child attachment, mother–child attachment, child self-esteem were all positively correlated with each other and in the hypothesized direction (*ps* < 0.01). Furthermore, family cohesion/adaptability, father–child attachment, mother–child attachment, and child self-esteem were negatively associated with child depression (*ps* < 0.01). However, only child self-esteem was significantly and negatively related to child aggressive behavior (*p* < 0.01). Additionally, the demographic variables (i.e., children’s gender, paternal age, paternal education, maternal age, maternal education, and family monthly income) were related to several of the observed variables. Thus, these demographic variables were examined as covariates in later data analyses.

### 3.2. The Mediating Roles of Mother–Child and Father–Child Attachment and Child Self-Esteem

**Figure 2:**
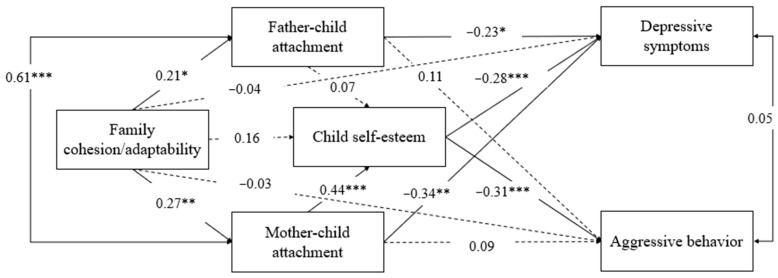
Results Model of Multilevel Family Factors and Emotional and Behavioral Problems of Children with ODD. Note: These are standardized model results and children’s gender and age, parents’ age and education, and family monthly income (not shown) were included as covariates in the model. * *p* < 0.05, ** *p* < 0.01, *** *p* < 0.001.

The final model with standardized path coefficients is presented in Figure 2. The model fit the data very well, *χ*^2^ (21) = 22.23, *p* = 0.39, CFI = 0.99, TLI = 0.99, RMSEA = 0.02 (90% CI = [0, 0.07]), and SRMR = 0.03. The direct effects of multilevel family factors on emotional and behavioral problems in children with ODD is displayed in Table 2. The results showed that family cohesion/adaptability was not directly associated with child depression (*β* = −0.04, *p* = 0.45) or aggressive behavior (*β* = 0.03, *p* = 0.64). The direct relation between mother–child attachment and father–child attachment and child depression (*β*_mother–child attachment_ = −0.34, *p* < 0.01; *β*_father–child attachment_ = −0.23, *p* < 0.05) were significant, while mother and father attachments were not related to child aggressive behavior (*β*_mother–child attachment_ = 0.09, *p* = 0.39; *β*_father–child attachment_ = 0.11, *p* = 0.25). Furthermore, a significant association emerged between child self-esteem and child depression (*β* = −0.28, *p* < 0.001) and aggressive behavior (*β* = −0.31, *p* < 0.001).

The indirect effects for this mediation model is displayed in Table 3. Family cohesion/adaptability was indirectly associated with child depression (*β* = −0.03, *p* < 0.05, 95%CI = [−0.06, −0.01]) and aggressive behavior (*β* = −0.04, *p* < 0.05, 95%CI = [−0.07, −0.01]) via mother–child attachment and child self-esteem in sequence. 

Mother–child attachment also played a significant role as a mediator in the association between family cohesion/adaptability and child depression (*β* = −0.09, *p* < 0.05, 95%CI = [−0.17, −0.01]). However, father–child attachment did not mediate the links between family cohesion/adaptability and child depression (*β* = −0.05, *p* = 0.11, 95%CI = [−0.11, 0.01]) or between family cohesion/adaptability and aggressive behavior (*β* = 0.03, *p* = 0.33, 95%CI = [−0.02, 0.07]).

Additionally, child self-esteem partially mediated the links between mother–child attachment and child depression (*β* = −0.12, *p* < 0.01, 95%CI = [−0.20, −0.05]), and completely mediated the relationship between mother–child attachment and child aggressive behavior (*β* = −0.14, *p* < 0.01, 95%CI = [−0.22, −0.05]). However, child self-esteem did not mediate the link between father–child attachment and child depression (*β* = −0.02, *p* = 0.43, 95%CI = [−0.07, 0.03]) or between father–child attachment and aggressive behavior (*β* = −0.02, *p* = 0.42, 95%CI = [−0.07, 0.03]).

The model as a whole account reached 54% of the variance in child depression, 30% of the variance in child self-esteem, 24% of the variance in child aggressive behavior, 7% of the variance in mother–child attachment, and 5% of the variance in father–child attachment, ranging from large to small.

## 4. Discussion

The present study aimed to examine the association of multilevel family factors with the emotional and behavioral problems of children with ODD. Our findings indicated that family cohesion/adaptability at system level was indirectly related to emotional and behavioral problems via the mother–child attachment at a dyadic level and child self-esteem at an individual level in sequence. This finding extended previous findings by demonstrating that the multilevel family model could also explain the effects of the system, dyadic, and individual level family factors on the emotional and behavior problems of children with ODD. Regarding dyadic mother– and father–child attachment, we found that only mother–child attachment mediated the association between family cohesion/adaptability and child depression, while father–child attachment was not a significant mediator. These results suggested that mother–child attachment within Chinese families impacts child depressive symptom outcomes to a greater extent. Moreover, child self-esteem partially mediated the link between mother–child attachment and child depression, and completely mediated the relationship between mother–child attachment and child aggressive behavior. This finding highlighted the importance of carefully considering the role of self-esteem as an individual child characteristics on the development of emotional and behavioral problems. Taken together, the findings of the present study provided unique insights into explaining how multilevel family factors differently and uniquely relate to emotional and behavioral problems in children with ODD. Furthermore, the study’s results could contribute to the development of educational guidance for families with children who have emotional and behavioral problems.

Our findings that family cohesion/adaptability, at the system level factor, was indirectly linked to emotional and behavioral problems through mother–child attachment and child self-esteem were in line with previous findings [38,39] and consistent with person–context interaction theory and multilevel family factors model. In previous studies conducted in Western cultures, family cohesion, a distal family factor, was found to be associated with child behavioral problems through more proximal factors, such as parent–child interactions [62]. From our findings, we could postulate that findings generalize to families in Mainland China. Indeed, within the cohesive family environment, there were more positive interactions between parents and children, which contributed to a higher quality of parent–child attachment and higher levels of child self-esteem. The higher quality of parent–child attachment and higher levels of child self-esteem appeared to protect children with ODD from further developing emotional and behavioral problems. The findings also validate the ancient Chinese proverb, “A harmonious family brings prosperity”. Cohesion and adaptability within the family would facilitate parent–child attachment and child development, even among children with ODD. The findings indicated the urgent need to understand the emotional and behavioral problems of children with ODD within the broader family contexts, instead of focusing solely on one family factor. In terms of clinical practice, the findings highlighted the significance of a positive family environment for the development of children with ODD.

As hypothesized, mother–child attachment, a dyadic level family factor, was associated closely with child development within the family context. Our study found that mother–child attachments mediated the relationship between family cohesion/adaptability and child depression. However, the mediating role of father–child attachments was not significant. Thus, mother–child attachment, unlike father–child attachment, was a significant mediator in the relationships between system level family factors and the individual outcomes of children with ODD [38]. This conclusion is consistent with the dominant hypothesis [24] and the concept of the traditional division of the family roles [63]. Recently, as a result of social and cultural shifts, more fathers have progressively accepted greater family duties. However, under the traditional gender division of labor, mothers are still the primary caretakers, responsible for the children’s everyday lives and diverse socioemotional needs. This was particularly true for mothers of children with emotional and behavioral problems [48,63]. Fathers tend to take a secondary role in the family. Societal expectations and gender norms forced fathers to prioritize financial assistance. Due to this division of work, the mother–child attachment was more crucial to the development of the child than the father–child attachment. Additionally, this implicates that father–child and mother–child attachments were differentially related to child outcomes [64,65]. The father–child attachment was closely related to the social development of the child, while the mother–child attachment was mainly linked to the internal psychological outcomes of children, such as emotional problems [7]. These results all suggested that the mother–child attachment is a pivotal dyadic-level factor that linked with child emotional development, specifically in families with children identified with ODD. Thus, it is important that future research and home-based educational guidance for children with ODD should focus on family-related dyadic factors, such as mother–child attachment.

Results in the current study also indicated that child self-esteem mediated the spillover effect from mother–child attachment to the emotional and behavioral problems of children with ODD. This finding highlighted that child self-esteem, as an individual level factor, was an important pathway via which dyadic level factors exert function on child development. In fact, according to the sociometer hypothesis [66], self-esteem is a sociometer that is involved in the maintenance of interpersonal relations. Moreover, Leary (1990) proposed that individual self-esteem is associated with the evaluation of others given that the individual needs to be accepted in society. Children with ODD who experience lower levels of mother–child attachment might internalize negative perceptions of being rejected by their mothers, which might lead to lower self-esteem. Low self-esteem, in turn, can futher exacerbate emotional and behavioral problems in children [41,67]. Conversely, higher quality of mother–child attachment may contribute to a higher level of self-esteem in children, which can buffer against other emotional and behavioral problems. These findings point to the importance that child self-esteem played in the relationship between dyadic level factors (i.e., mother–child attachment) and child psychological outcomes. Importantly, it is worth noting that although children with ODD are more likely to exhibit emotional and behavioral problems, higher levels of self-esteem can improve the healthy development of children, and decrease the occurrence of emotional and behavioral problems. Taken together, these findings can help inform and improve services for both families with children with ODD and with emotional or behavioral problems.

### 4.1. Limitations and Future Prospects

Our findings should be interpreted in light of several limitations. First, our study predominantly focused on the hierarchy of family factors at different levels and their effects on problem behaviors in children with ODD, the mutual linkages are not further elaborated on in this study. Additionally, with a cross-sectional method, we could not infer causal relationships or examine reciprocal relationships. However, the interplay of multilevel family factors and the emotional and behavioral problems of children with ODD might initiate transactional feedback loops. Longitudinal research should underscore the reciprocal relationships between multilevel family factors and child ODD symptoms. Second, the data collected in the present study were based on self-reports from parents, class master teachers, and children, which could have biased our results. Future studies should aim to reduce the bias caused by self-report methods and prioritize multi-informant ratings to better capture the heterogeneity of family dynamics. A further limitation is that we did not eliminate the influence of the different survey methods utilized in Grades 1–2 and Grades 3–5. To properly reflect the developmental outcomes of young children, future research should embrace more objective approaches. Fourth, the current study only examined parent–child attachment at the dyadic level and child self-esteem at the individual factor. Observing both parent–child relationships and marital relationships on the dyadic level and both the child and parent factors on the individual level may offer a new and integrated perspective to explore the association. Another concern is that caution is needed when generalizing the results of our study across cultures or age groups. As was previously stated, the core family values of Chinese culture are distinct from those of Western civilizations [68]. Consequently, the specific links discovered between multilevel family factors and emotional and behavioral problems in children with ODD must be confirmed in Western countries.

### 4.2. Implications

Despite these limitations, the present study substantiated and enriched the multilevel family factors model [12,38] by considering mother–child and father–child attachments and child self-esteem concurrently. Findings in the current study contributed to our understanding of how multilevel family factors relate to the emotional and behavioral problems of children with ODD. For researchers and practitioners working with families that have children with ODD, attuning to the family environment (family cohesion and adaptability), parent–child attachment (particularly mother–child attachment) may help decrease the severity of child emotional and behavioral problems. Furthermore, child self-esteem, as a vital self-protective factor, should be emphasized as a mechanism that can help prevent emotional and behavioral problems in children with ODD.

## 5. Conclusions

The current study examined the associations between multilevel family factors (i.e., family cohesion/ adaptability at a system level, mother–child and father–child attachment at a dyadic level, and child self-esteem at an individual level) and emotional and behavioral problems among children with ODD in China. This study contributes to the research on the development of children with ODD who have comorbid emotional and behavioral problems through an examination of a theory-based model proposed by Lin et al. (2022) [12]. The results revealed that a system level factor (family cohesion/adaptability) was associated with child emotional and behavior problems indirectly through factors at the dyadic level (mother–child attachment) and the individual level (child self-esteem) in sequence. Mother–child, but not father–child, attachments, mediated the linkage between family cohesion/adaptability and the emotional problems of children with ODD. Moreover, child self-esteem mediated the association between mother–child attachment and child emotional and behavioral problems. These results underscored the significance of understanding the emotional and behavioral problems of children with ODD within the framework of the family, and more particularly, within the context of the multiple levels of family relationships. The research highlighted the need for practitioners to carefully consider the features of the systemic family and the unique relationship between multilevel family factors and child outcomes.

## Figures and Tables

**Table 1:** Means, Standard Deviations, and Correlations Among Study Variables.

	1	2	3	4	5	6	7	8	9	10	11	12	13
1. Children’s gender	1												
2. Children’s age	−0.01	1											
3. Paternal age	0.18 **	0.17 **	1										
4. Maternal age	0.15 *	0.23 **	0.80 **	1									
5. Paternal education	0.06	−0.25 **	0.24 **	0.19 **	1								
6. Maternal education	−0.06	−0.28 **	0.07	0.08	0.75 **	1							
7. Monthly income	0.23 **	−0.20 **	0.32 **	0.24 **	0.60 **	0.48 **	1						
8. Family cohesion/adaptability	−0.13	−0.10	−0.10	−0.05	0.23 **	0.20 **	0.17 *	1					
9. Father–child attachment	−0.11	−0.02	−0.02	0.01	0.08	0.17 *	0.12	0.22 **	1				
10. Mother–child attachment	−0.08	−0.03	−0.09	−0.04	0.09	0.21 **	0.10	0.27 **	0.70 **	1			
11. Child self-esteem	−0.20 **	−0.05	−0.08	−0.01	0.06	0.21 **	0.01	0.28 **	0.39 **	0.48 **	1		
12. Child depression	0.15 *	−0.02	0.04	−0.05	0.10	−0.04	0.09	−0.24 **	−0.58 **	−0.64 **	−0.58 **	1	
13. Aggressive behavior	0.34 **	0.04	0.28 **	0.28 **	0.21 **	0.13 *	0.25 **	−0.06	−0.03	−0.10	−0.20 **	0.21 **	1
Mean		9.60	38.43	36.66	3.87	3.74	2.79	121.06	23.88	25.74	30.53	36.06	17.90
SD		1.57	5.16	4.29	1.33	1.36	1.03	17.19	11.80	12.24	6.16	10.40	7.77

Note. Children’s gender was coded 1 for boy and 0 for girl. Parental education was measured on a 6-level categorical variable (1 = elementary school diploma, 6 = master’s degree). Family monthly income was measured on a 5-level categorical variable (1 = 2000 Chinese Yuan or less, 5 = 30,000 Chinese Yuan or more). * *p* < 0.05, ** *p* < 0.01.

**Table 2:** Direct Effects of Multilevel Family Factors on Child Outcomes.

Path	*Β*	*SE*	*p*	95%CI
Child depression as outcome				
family cohesion/adaptability → child depression	−0.007	0.03	0.91	[−0.06, 0.07]
father–child attachment → child depression	−0.25	0.10	0.046	[−0.45, −0.03]
mother–child attachment → child depression	−0.36	0.11	0.009	[−0.51, −0.07]
child self-esteem → child depression	−0.31	0.14	<0.000	[−0.82, −0.23]
Child aggression as outcome				
family cohesion/adaptability → child aggression	0.03	0.03	0.64	[−0.08, 0.05]
father–child attachment → child aggression	0.03	0.07	0.21	[−0.06, 0.24]
mother–child attachment → child aggression	−0.05	0.08	0.55	[−0.11, 0.20]
child self-esteem → child aggression	−0.13	0.10	0.001	[−0.60, −0.16]

Note. Covariates are children’s gender and age, parents’ age and education, family monthly. Income (not shown).

**Table 3:** Indirect Effects for Mediation Models.

Path	*β*	*SE*	*p*	95%CI
Child depression as outcome				
family cohesion/adaptability → mother–child attachment → child depression	−0.09	0.04	0.04	[−0.17, −0.01]
family cohesion/adaptability → father–child attachment → child depression	−0.05	0.03	0.11	[−0.11, 0.01]
family cohesion/adaptability → child self-esteem → child depression	−0.04	0.03	0.15	[−0.10, 0.01]
mother–child attachment → child self-esteem → child depression	−0.12	0.04	0.002	[−0.20, −0.05]
father–child attachment → child self-esteem → child depression	−0.02	0.03	0.45	[−0.07, 0.03]
family cohesion/adaptability → mother–child attachment → child self-esteem → child depression	−0.03	0.02	0.03	[−0.06, −0.01]
family cohesion/adaptability → father–child attachment → child self-esteem → child depression	−0.004	0.01	0.54	[−0.02, 0.01]
Child aggression as outcome				
family cohesion/adaptability → mother–child attachment → child aggression	0.03	0.03	0.45	[−0.04, 0.09]
family cohesion/adaptability → father–child attachment → child aggression	0.03	0.03	0.35	[−0.02, 0.07]
family cohesion/adaptability → child self-esteem → child aggression	−0.05	0.03	0.08	[−0.10, 0.01]
mother–child attachment → child self-esteem → child aggression	−0.13	0.05	0.003	[−0.22, −0.05]
father–child attachment → child self-esteem → child aggression	−0.02	0.03	0.46	[−0.07, 0.03]
family cohesion/adaptability → mother–child attachment → child self-esteem → child aggression	−0.04	0.02	0.03	[−0.07, −0.01]
family cohesion/adaptability → father–child attachment → child self-esteem → child aggression	−0.01	0.01	0.54	[−0.02, 0.01]

Note. Covariates are children’s gender and age, parents’ age and education, family monthly income (not shown).

## Data Availability

The datasets generated during and/or analyzed during the current study are available from the corresponding author on reasonable request.
